# Going through the motions: biasing of dynamic attentional templates

**DOI:** 10.1037/xge0001665

**Published:** 2025-01

**Authors:** Sage E.P. Boettcher, Anna C. Nobre

**Affiliations:** 1 Department of Experimental Psychology, University of Oxford, Oxford, United Kingdom; 2 Oxford Centre for Human Brain Activity, Wellcome Centre for Integrative Neuroimaging, Department of Psychiatry, University of Oxford, Oxford, United Kingdom; 3 Wu Tsai Institute, Yale University, New Haven, CT, United States

**Keywords:** Visual Search, Attentional template, Memory, Attention

## Abstract

Attention must coordinate with memory to actively anticipate sensory input and guide action. Memory content may be biased away from veridical when it is functionally adaptive. So far, research has considered the biasing of still features in static displays. It is unknown whether the biasing of attentional templates can functionally adapt dynamic stimuli to facilitate search when targets and distractors compete within temporally extended contexts. Biasing of dynamic templates would require learning and modulatory mechanisms capable of abstracting over space and time to guide perception. Four experiments used a novel dynamic visual search task combined with a memory probe to test whether dynamic attentional templates can be biased. In Experiments 1-3, participants searched for a moving target among distractors that systematically moved either clockwise or counterclockwise relative to the target. On memory probe trials, participants recalled the target direction as biased away from the distractors. The distortion bias was adaptively changed (Experiment 2), grew over time (Experiment 2), and occurred even when motion direction was not the target-defining feature (Experiment 3). Experiment 4 manipulated the speed of targets and distractors to test the generalisability of the findings. Participants searched for a target of a given speed among faster or slower distractors. Memory probing revealed that participants remembered the target speed as biased away from that of distractors. Across different tasks, the magnitude of the biasing correlated positively with search performance. Our findings provide compelling evidence that dynamic stimulus attributes in attentional templates can become functionally biased when adaptive.

## Introduction

Natural behaviour depends on attentional mechanisms successfully coordinating with memory to anticipate sensory input and guide our actions (Nobre & Stokes, 2019). Attention is often guided by internal representations derived from our past experiences with external stimulation. These *attentional templates* exert two important potential roles in models of visual search (Carlisle et al., 2011; Duncan & Humphreys, 1989; Geng & Witkowski, 2019; Wolfe, 2021). First, they help guide attention to select candidate targets. Second, they help determine whether the selected information is task-relevant (Wolfe, 2021).

Classically, it was assumed that attentional templates represent the relevant attributes veridically, with the same qualities with which these attributes are perceived. However, a growing body of evidence suggests this need not be the case (Becker et al., 2010, 2013; Boettcher, van Ede, et al., 2020; Geng & Witkowski, 2019; Navalpakkam & Itti, 2007; Scolari & Serences, 2009; Yu & Geng, 2019). For example, Yu and Geng (2019) showed that features of target templates are asymmetrically biased away from those of expected distractors when this distortion has higher utility in guiding search. Participants searching for blue-green targets among bluer distractors recalled their target as greener than the true hue. Similarly, beyond target templates for visual search, there is increasing acceptance that information is not necessarily held veridically within working memory. Instead, representations tend to be adaptive and capture both groupwise statistics and individual features (Bae & Luck, 2017; Brady & Alvarez, 2011; Chunharas et al., 2022; Scotti, Hong, Golomb, et al., 2021; Scotti, Hong, Leber, et al., 2021). The exact mechanisms leading to distortions of memory content in attentional templates are still debated, with ongoing discussion concerning the degree to which low-level *perceptual* effects contribute to the effect (Hamblin-Frohman & Becker, 2021; Yu et al., 2022). However, irrespective of the exact mechanisms, ample evidence points to representations *in memory* used for behavioural guidance diverging from faithful reproductions of the external stimulation when adaptive (Becker et al., 2010, 2013; Boettcher, van Ede, et al., 2020; Geng & Witkowski, 2019; Kerzel, 2020; Navalpakkam & Itti, 2007; Scolari & Serences, 2009; Yu et al., 2022; Yu & Geng, 2019).

Most standard laboratory tasks exploring the role of attentional templates have used static displays and stimulus attributes. However, real-world attention unfolds in dynamic contexts as we move through the world and the world changes around us. For example, imagine trying to find your friend in a pack of runners or trying to find the football amid moving players on a field. In these colloquial search examples, our attentional template may contain static features – such as colour, shape, and size – but also dynamic features – such as the motion direction or speed of our target. Dynamic features that integrate across time and space, such as motion direction and speed, have been shown to guide attention within the context of visual search (Driver et al., 1992; Horowitz et al., 2007). We have also recently shown that spatiotemporal regularities can be learned and used to guide attention during dynamic visual searches (Boettcher et al., 2021; Shalev et al., 2022). Such findings reaffirm the importance of the often-overlooked temporal dimension of our attentional system (Nobre & van Ede, 2017, 2023).

Extending the template-biasing notion to dynamic stimuli poses theoretically important and non-trivial challenges to the brain. Biasing dynamic templates requires more than just altering one brain state associated with the stable feature(s) of a target. Instead, the biasing must modify a trajectory of states that come to differentiate targets from distractors. With dynamic stimuli, it is necessary to integrate signals over time and ultimately relate these signals to similarly dynamic representations of other stimuli. When dynamic stimuli lack consistent starting or ending spatial locations or temporal intervals, there is no simple feature that can serve as a spatial or temporal anchor for modulation. Biasing of dynamic templates would highlight a more general principle of attentional templates which extends beyond classically tested static feature.

Bringing together observations of template distortions and the importance of dynamic features and extended contexts for attention, we ask whether dynamic stimulus attributes can be distorted from the veridical stimulation when it is helpful to do so. Such biasing would imply flexibility that unfolds across time to extract and utilise functionally relevant content to guide behaviour proactively. Across four studies, we probed participants about the target information they held in memory to complete a dynamic visual search task. In a preview of our results, we found systematic and adaptive biasing of dynamic attributes of motion direction (Experiments 1-3) and speed (Experiment 4).

## Experiment 1: Biasing motion direction in dynamic attentional templates

### Methods

All experimental procedures were reviewed and approved by the Central University Research Ethics Committee of the University of Oxford. Participants were briefed, completed the experiment, and were debriefed online. The experimental task was generated using PsychoPy (Peirce et al., 2019) and was hosted on Pavlovia (http://pavlovia.org). Briefings were carried out using Qualtrics (http://qualtrics.com). Procedures for setting participant numbers according to statistical power, recruiting and compensating participants, and checking data quality for inclusion in the final analysis were the same across all experiments.

#### Transparency and Openness

Below, we report details on the power analysis for which we based our sample size, data exclusion criteria, and all measures within the experiment. The data, analysis code, experimental code, and additional resources can be accessed https://osf.io/4mv9g/ (Boettcher & Nobre, 2023). The experiment was not preregistered.

#### Participants

Participants were recruited via Prolific Academic (https://www.prolific.co/), a platform for online participant recruitment that is well suited for conducting web-based academic research (Palan & Schitter, 2018; Sauter et al., 2020). Pre-screening criteria ensured participants were between 18 and 40 years old, fluent in English, had normal or corrected vision and colour perception, and had completed a minimum of 10 studies on prolific with a study approval rating above 90%. Participants were asked to report their gender, age, and handedness in free-response boxes. All participants provided written consent prior to participation and were compensated at a rate of £7.50 per hour.

A total of 223 participants were recruited. Of these, after data-quality checks, data from 168 participants were retained for analysis (see *Data Preprocessing*). The analysis included 84 participants assigned to the clockwise-distractors condition (see below). They were aged between 18 and 40 (M = 26.1, SD = 5.59), 26 were female, 58 were male, 77 were right-handed, and 7 were left-handed. The other 84 participants were in the counterclockwise-distractors condition. They were aged between 18 and 38 (M = 24.1, SD = 4.84), 29 were female, 55 were male, 72 were right-handed, and 12 were left handed. The final sample size was determined *a priori* using a power analysis in G*Power (Faul et al., 2007) targeted on the detection of a medium effect size for the planned between-subjects comparison.

#### Task and Procedure

All experimental procedures were reviewed and approved by the Central University Research Ethics Committee of the University of Oxford. Participants were briefed, completed the experiment, and were debriefed online. The experimental task was generated using PsychoPy (Peirce et al., 2019) and was hosted on Pavlovia (http://pavlovia.org). Briefings were carried out using Qualtrics (http://qualtrics.com).

Before starting the experiment, participants were shown an overview of the task and a video with examples of several trials. Their task was to find a moving target dot, defined by a particular direction of motion, among moving distractor dots. Participants were shown the target direction (either 130° or 310°; randomly assigned between participants) that they had to retain in memory and use for identifying targets throughout the experiment. These motion directions were chosen so that both upward and downward motions were represented and distractors (whose directions were chosen relative to the target) would not cross a cardinal axis.

**Figure 1:**
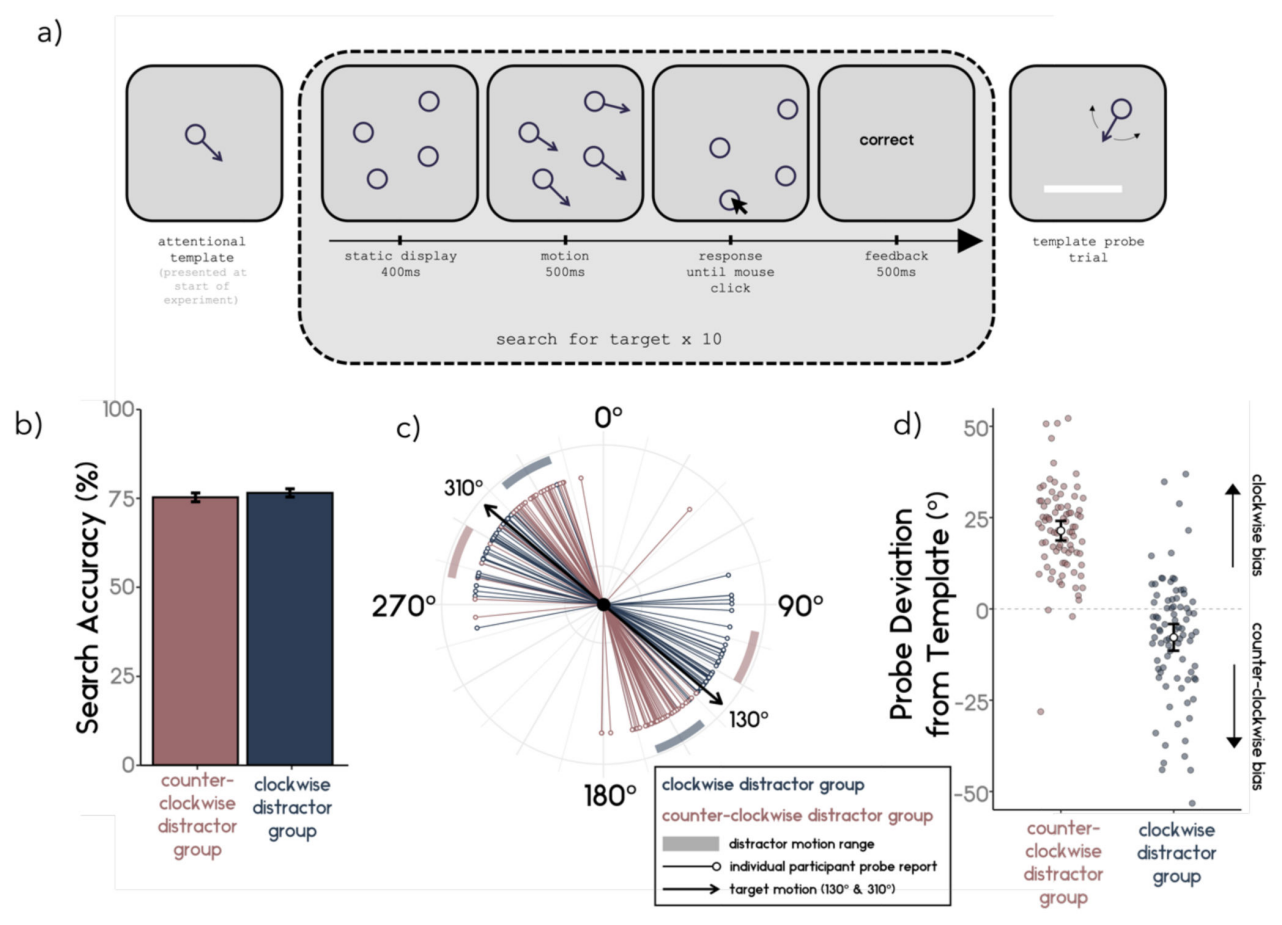
a) Schematic of Experiment 1. Participants were presented with the target direction of motion (attentional template) at the start of the experiment. During search trials, participants searched for their target motion among distractor dots moving either clockwise or counterclockwise relative to the target (balanced across participants). At the end of each search block, participants completed a probe trial in which they reported their template direction of motion by rotating a dial. b) Participants’ search performance did not differ across the distractor groupss c) Participant probe reports. Target motion was either 130° or 310° (black arrows) and the possible distractors encountered during the search were either clockwise (blueish shaded region) or counterclockwise (reddish shaded region) relative to the target motion. Participants’ average probe reports are plotted as thin coloured lines. Participants who encountered clockwise distractors on average reported their target as more counterclockwise, while those who encountered counterclockwise distractors reported the target as more clockwise. d) Average probe deviation from template. Individuals’ average deviation during probe trials from the true target motion direction for participants from the counterclockwise (reddish) and clockwise (blueish) are plotted as small dots. Positive (negative) deviations indicate that the participant reported the probe as more clockwise (counterclockwise) than the target truly was. The average deviation for each group is represented as a white dot. Error bars represent the 95% confidence interval around the mean.

Figure 1a shows a schematic of the task. Broadly, the experiment contained two types of trials: search trials and probe trials. Each block consisted of ten successive search trials followed by a single probe trial. Participants first completed one practice block before continuing to ten experimental blocks.

On each search trial, participants viewed an array of four identical dots presented at pseudo-random locations on the screen. Dot outlines were dark purply blue (RGB 45, 42, 76), and the internal colour matched the grey background of the display (RGB 128, 128, 128). Each stimulus had a diameter of .1 normalised units relative to the size of the display window, which ranged from -1 to +1 in both the x and y directions. The size of the full display window depended on the screen participants were using.

After a delay of 400 ms, each stimulus began to move from its starting position following a linear path at a rate of .6 normalised units/second. The stimuli moved for a total of 500 ms. Target dots always moved along the specified target motion direction throughout the experiment (130° or 310°). The motion paths for each of the distractor dots deviated radially from the target direction by either 10°, 20°, or 30°. The exact combination of distractors was chosen randomly, with replacement, on each trial.

The primary experimental manipulation was the clockwise vs. counterclockwise deviation of the motion directions of distractors relative to the target. Participants assigned to the clockwise distractor group were only exposed to distractors that moved +10°, +20°, or +30° (clockwise) from the target. Participants in the counterclockwise group only encountered distractors that moved -10°, -20°, or -30° (counterclockwise) from the target. After the stimuli stopped moving, participants clicked on the target and then received feedback onscreen (either ‘correct’ or ‘incorrect’) for a 500-ms period. After a random uniformly sampled delay between 300 and 800 ms, the next trial began.

During probe trials, participants were asked to reproduce the direction of motion of their target. These trials were used as a measure of the attentional template in memory. Specifically, participants reported their target direction using a radial dial, which appeared at a random location on the screen. A line radiated from the centre of the dial, rotating according to the location of the participant’s cursor. Participants chose the final dial location to match the motion direction of the target. The report dial appeared at random locations across the screen to minimise spatial biases and systematic errors in reports (e.g., caused by participants simply clicking at a single location onscreen). Participants were required to complete their report within 4000 ms, as indicated by a white progress bar at the bottom of the screen. Participants did not receive any feedback in the probe trials.

#### Analysis

Data analyses were carried out in R Studio (Rstudio Team, 2019). Data were first pre-processed and cleaned according to an *a priori* pipeline to ensure the highest level of data quality. Specifically, slow search trials were initially removed from the data set – trials in which the RT exceeded 5000 ms or was 2.5 SD above the individual mean. A cut-off was set to remove participants with more than 10% slow searches from the analysis, but this did not result in the loss of any datasets. The next cut-off was to remove participants with poor search performance (< 50% correct; chance was 25%). This resulted in the loss of 49 datasets. Probe trials were removed if participants timed out (i.e., took longer than 4000 ms to respond) or reported a probe deviation greater than 2.5 SD from their mean. As participants only completed 10 probe trials, datasets with a loss of more than 2 probe trials (20%) were discarded. This resulted in the loss of one additional dataset. Additionally, participants with an overall probe deviation greater than 2.5 SD from the grand mean were considered outliers and their data sets were removed, resulting in the additional loss of two datasets. Finally, in a debriefing, after the task finished, participants were asked if they believed their data should be analysed and were informed that their answer would not affect their payment. Fifteen participants indicated that they did not believe their data should be included. These data sets were also removed from the final data set if they had not already been removed for other reasons. In total, 55 datasets were removed from the final sample resulting in a final sample size of 168 participants. Our main results did not depend on these data cleaning procedures (Supplementary Figure 1).

First, we checked whether search performance differed across the two distractor conditions by conducting a Welch’s two-sample t-test comparing the average search accuracy for participants with clockwise vs. counterclockwise distractors. We chose search accuracy as the main metric of search performance because response times in our study were not reliably informative. Response times could be decoupled from when participants found the target because they waited until the stimuli stopped moving before making their response.

Our main analysis focused on the probe trials. The dependent variable of interest was the average probe deviation between the reported probe direction of motion and the actual target direction of motion (using the shortest distance along the circle). Average probe deviation was calculated per participant and collapsed across the two possible target directions (130° & 310°). An average deviation of zero would indicate no bias. An average positive deviation would indicate a bias in the clockwise direction, whereas an average negative deviation would indicate a bias in the counterclockwise direction. The main independent variable was distractor group (clockwise vs. counterclockwise), which was manipulated between participants. Our main analysis consisted of a Welch’s two-sample *t-test* comparing the average probe deviation for participants with clockwise vs. counterclockwise distractors. This was followed by two one-sample *t-tests* comparing the mean in probe deviation in each group against zero. All statistical tests were run using the *lsr package* in R (Navarro, 2015). Throughout, *Cohen’s d* serves as a measure of the effect size.

Finally, we wished to gain insight into the relationship between probe deviation and search performance. Specifically, we calculated each participant’s average search accuracy for a block of trials as well as their probe deviation in the corresponding block. Probe deviation was transformed such that positive values always indicated a high deviation independent of the distractor context. We conducted our statistical test in two manners. First, we collapsed across all participants and conducted a Spearman’s Rank correlation test. Next, to probe whether this correlation remained significant at the individual subject level, we calculated each participant’s correlation coefficient across blocks (i.e., 1 point per block representing average search performance and probe deviation on that block) and then conducted a one-sample t-tests comparing the mean of the participants’ correlation coefficients against zero. A significantly positive value would indicate that participants showed a significant positive relationship between search performance and probe deviation.

## Results

### Search Performance

Figure 1b *shows the average search accuracy for participants with counter- and clockwise distractors. The average search accuracy did not differ between the two groups (t*_(107.66)_ = -0.16, *p* = .872, *d* = .031; clockwise distractor group M = 76.53, SD = 10.6; counter-clockwise distractor group M = 71.99, SD = 11.3).

#### Probe Deviation

Figures 1c & 1d show that distractor group had a significant effect on probe deviation (*t*_(152.23)_ = 12.82, *p* < .001, *d* = 1.98). The average probe deviation for the counterclockwise distractor group was biased in the clockwise direction (M = 21.41; SD = 12.35) (*t*_(83)_ = 15.89, *p* < .001, *d* = 1.73), whereas the average probe deviation for the clockwise distractor group was significantly biased in the counterclockwise direction (M = -7.8; SD = 16.84) (*t*_(83)_ = -4.24, *p* < .001, *d* = .46).

#### Relationship between search performance and probe deviation

We found a significant relationship between probe deviation and search accuracy (Supplementary Figure 2). There was a positive correlation (r = .231, p < .001) between the two variables, indicating higher search accuracy was associated with greater probe deviation. Moreover, when the correlation coefficient was calculated per participant (across blocks), we found that the average correlation coefficient is significantly greater than zero (t_(167)_ = 4.02, p < .001, d = .31; M = .115, SD = .370)

## Discussion

The results of Experiment 1 showed that participants can search for dynamic targets according to motion direction and that the distractor context strongly biases the representation of the target motion direction. Rather than reporting the veridical target direction, participants reported targets as being biased away from the distractor directions.

That is, participants who encountered clockwise distractors reported the target as more counterclockwise, and participants who encountered counterclockwise distractors reported the target as more clockwise. Further, we find that when participants showed a greater bias in their report of their template, they were also significantly more accurate when searching during that block.

The findings provide initial evidence that participants update a dynamic target representation to achieve a better functional separation between the target and potential distractors. However, the results leave unclear how flexible such a system may be. The current experiment relied on a between-subjects design, in which participants encountered only one distractor context within the experiment, without the need to adjust biases flexibly. Experiment 2 tested whether the biasing of dynamic target representations occurs when the distractor context is more volatile. We tested whether participants flexibly update the representation of a dynamic target and, in turn, whether search performance is modulated accordingly. Moreover, because participants in Experiment 1 remained consistently within the same distractor context, the experiment was not well suited for understanding how such a bias emerges with time. By alternating distractor contexts, Experiment 2 additionally investigated how distortion biases evolve over time.

## Experiment 2: Flexible motion-direction biases in attentional templates

### Methods

#### Transparency and Openness

Like in Experiment 1, we report details on the power analysis for which we based our sample size, data exclusion criteria, and all measures within the experiment. The data, analysis code, experimental code, and additional resources can be accessed https://osf.io/4mv9g/ (Boettcher & Nobre, 2023). The experiment was not preregistered.

#### Participants

A total of 68 participants were recruited using Prolific Academic (https://www.prolific.co/). All participants provided written consent prior to participation and were compensated at a rate of £7.50 per hour. The pre-processing excluded 14 participants, leaving 54 participants in the final analyses. The final sample size was determined *a priori* using a power analysis in G*Power (Faul et al., 2007) targeted on the detection of a medium effect size. In the remaining set, participants were between 19 and 40 years old (M = 28.24, SD = 6.13). 18 participants reported their gender as female, 35 reported their gender as male, and 1 as non-binary. 46 participants were right-handed, while 8 were left handed.

#### Task and Procedure

**Figure 2:**
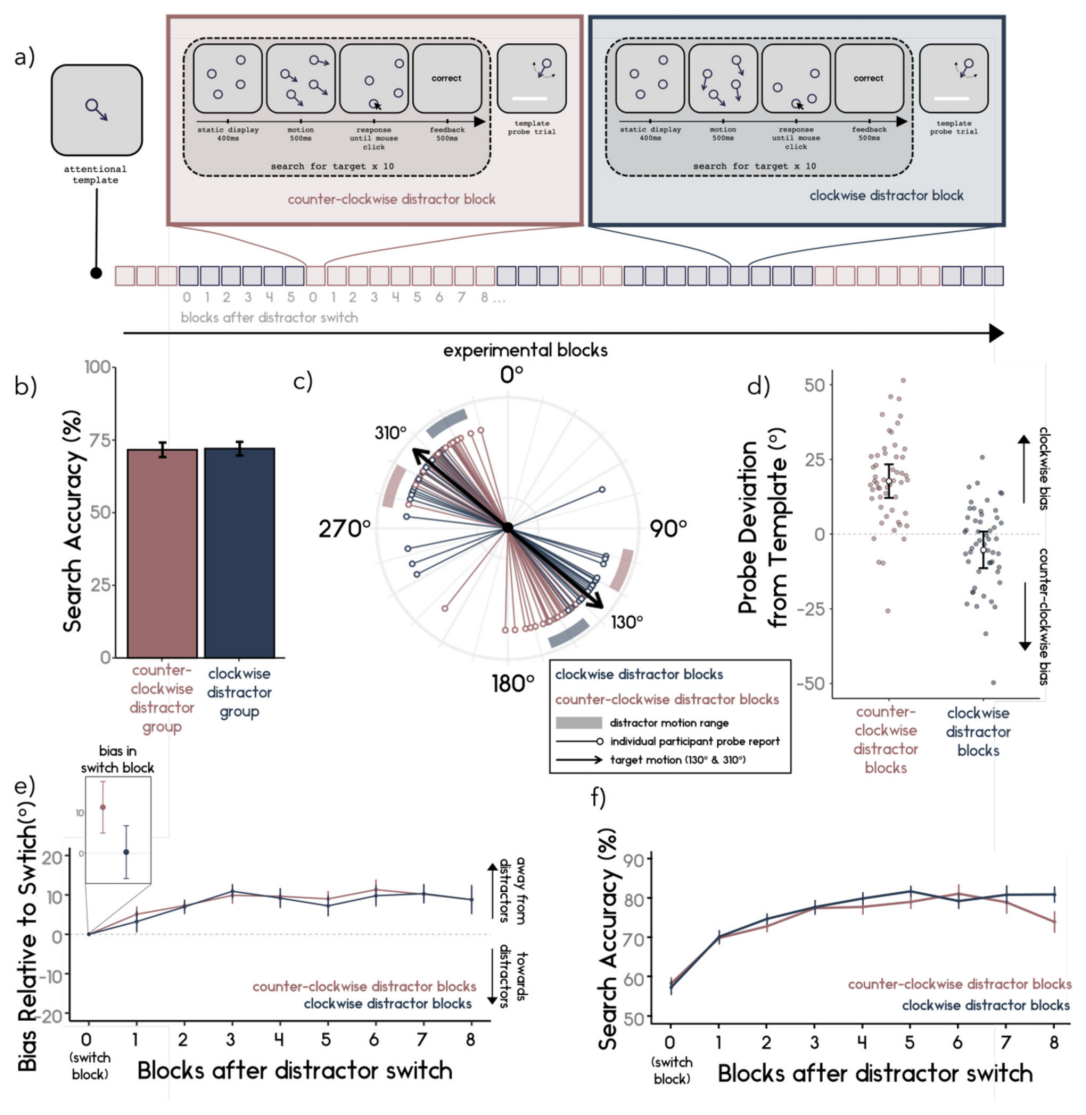
a) Schematic of Experiment 2. Participants were presented with the target direction of motion (attentional template) at the start of the experiment. The distractor condition now changed within participants between blocks. Participants completed in total 42 blocks (21 clockwise distractor blocks and 21 counterclockwise distractor blocks). Blocks were organised in runs of three successive blocks of the same type, after three blocks of the same type, the next run (clockwise or counterclockwise) was randomly chosen. b) Participants’ search performance did not differ in the two distractor conditions. c) Participants’ average probe reports are plotted as thin coloured lines. Target motion was either 130° or 310° (black arrows), and the possible distractors encountered during the search were either clockwise (blueish shaded region) or counterclockwise (reddish shaded region) relative to the target motion. d) Average probe deviation from template. Individuals’ average deviation during probe trials from the true target motion direction for the counterclockwise (reddish) and clockwise (blueish) blocks are plotted as small dots. Positive (negative) deviations indicate that the participant reported the probe as more clockwise (counterclockwise) than the target truly was. The average deviation for each group is represented as a white dot. Error bars represent the 95% confidence interval around the mean. e) The average bias per block relative to the switch block. Participants’ average bias on the switch block was used as a baseline. The inset shows the average bias on the switch blocks for each condition. The average change in bias (with positive values representing a bias away from distractors and negative values representing a bias toward distractors) was calculated for every block following a switch. Error bars here represent the standard error of the mean. f) Search accuracy relative to the switch block. Participants’ performance in the search task increased with the blocks from the switch.

Experiment 2 broadly followed the procedures from Experiment 1 with minor changes to test the flexibility of biases within dynamic target representations. A summary of the task is shown in Figure 2a.

As in Experiment 1, participants searched for a moving target dot defined by a particular direction of motion (either 130° or 310°, randomly assigned between participants). The stimulus parameters (colour, size, motion onset time, speed, duration, angular distractor

deviations, and feedback presentation) were the same as in Experiment 1. Participants completed 42 experimental blocks. Each block consisted of ten search trials followed by a probe trial. At the start of the experiment, participants were shown an overview of the task, their target direction of motion, and a video with examples of several trials, and given an opportunity to practice.

The main experimental manipulation, which differed from Experiment 1, was that the direction of distractor deviation varied between blocks. Participants completed 21 blocks with clockwise distractors and 21 blocks with counterclockwise distractors. Blocks were organised in runs of three successive blocks of the same type. After three blocks of the same type, the next run (clockwise or counterclockwise) was randomly chosen. We used a minimum run length of three blocks to test the time course for updating dynamic templates across the experiment.

During probe trials, participants reported their target direction using a radial dial like in Experiment 1.

#### Analysis

Data were first pre-processed and cleaned according to a pipeline matching that of Experiment 1. Search trials in which the RT exceeded 5000 ms or was 2.5 SD above the individual mean were removed. No participants were removed for having more than 10% slow searches. Eleven datasets were removed for poor search performance (< 50% correct). Probe trials were removed if participants timed out (i.e., took longer than 4000 ms to respond) or deviated more than 2.5 SD from the participant’s mean. No participants were removed for having more than 20% of probe trials discarded. One participant was removed for having an overall probe deviation greater than 2.5 SD from the grand mean. During debrief, two participants indicated their data should not be included. In total 14 datasets were removed from the final sample resulting in a final sample size of 54 participants.

We initially checked whether search performance differed across the two distractor conditions by conducting a repeated-measures *t-test* comparing the average search performance for clockwise and counterclockwise blocks.

Like Experiment 1, we were primarily interested in participants’ reports in the probe trials across the two block types (clockwise and counterclockwise). The main analysis consisted of a repeated-measures *t-test* comparing the average probe deviation for clockwise distractor blocks with counterclockwise distractor blocks. Two one-sample *t-tests* subsequently compared the mean in probe deviation in each group against zero. These statistical tests were run using the *lsr package* in R (Navarro, 2015), throughout, we report *Cohen’s d* as a measure of effect size.

To understand whether the bias associated with the distractor context builds with time we considered the independent variable *time after distractor switch*. As a dependent variable, we used a measure of bias (away or toward) relative to the switch block. To calculate this measure, we first calculated the average bias for each participant in clockwise and counterclockwise blocks for the *0-time after distractor switch block* (i.e., the first block of type X following a series of blocks of type Y). This was considered the baseline measure of bias (the average values for this baseline measure can be found in the inset in Figure 2e). We then subtracted this baseline measure of bias from the average probe deviation per block. Finally, we transformed this measure into an absolute measure of deviation such that positive values represent a bias *away* from the distractors and negative values represent a bias *towards* distractors.

We ran a linear-mixed effect model using the *lme4 package* (Bates et al., 2015). The model was fitted with the maximum likelihood criterion and here we report the β with the *t*-statistic and apply a two-tailed criterion corresponding to a 5% error criterion for significance. The *p*-values were calculated with Satterthwaite’s degrees of freedom method using the *lmerTest package* (Kuznetsova et al., 2017). *Time after distractor switch* and *condition* were used as predictors of *relative towardness. Time after distractor* was centered and entered the model as a continuous predictor, *condition* was modelled using sum contrasts such as the intercept represents the grand mean. All models used the maximal random-effects structure (Barr et al., 2013), with the participant’s intercepts and the by-participant slope for *condition* and *time after distractor*.

To test whether the search performance was also affected by the variable search displays, we repeated the above analysis with search accuracy as the dependent variable. Here, generalised linear mixed-effects models (GLMMs) with a binomial distribution were used to analyse search accuracy as a binary response. We report the regression coefficients β (as represented as logits) with the z statistic and we use a two-tailed 5% error criterion for significance. The p-values are based on asymptotic Wald tests.

To test the relationship between search accuracy and probe deviation, we again calculated each participant’s average search accuracy as well as their probe deviation in the corresponding block. Probe deviation was transformed such that positive values always indicate a high deviation no matter the distractor context. Like in Experiment 1, we tested for a significant correlation between these variables both across and within participants.

## Results

### Search Performance

Figure 2b *shows the average search accuracy for participants with counter- and clockwise distractors. The average search accuracy did not differ between the two groups (t*_(53)_ = -.21, *p* = .832, *d* = .029; clockwise distractor group M = 72.0, SD = 12.43; counter-clockwise distractor group M = 71.6, SD = 13.15).

#### Probe Deviation

The main experimental question was whether the distractor context flexibly biased the probe report when it changed within the experiment (Figures 2c & 2d). We found that distractor context (clockwise vs. counterclockwise blocks) had a significant effect on probe deviation (*t*_(53)_ = 6.32, *p* < .001, *d* = .86). The average probe deviation for the counterclockwise distractor blocks was biased in the clockwise direction (M = 17.73; SD = 14.54) (*t*_(53)_ = 8.96, *p* < .001, *d* = 1.22), whereas the average probe deviation for the clockwise distractor blocks was significantly biased in the counterclockwise direction (M = -5.28; SD = 15.54) (*t*_(53)_ = -2.45, *p* = .018, *d* = .33).

The measures of how biases away from distractors changed over blocks (Figure 2e) showed that the time from switch block was a significant predictor of relative deviation (β = .98, *SE* = .23, *t* = 4.34, *p* < .001). In contrast, distractor block type (clockwise vs. counterclockwise) was not a significant predictor of the magnitude of the relative deviation (β = .45, *SE* = .85, *t* =.54, *p* = .60). Block type and time did not interact significantly (β = .07, *SE* = .18, *t* = .40, *p* = .69). The results indicate that the repulsive bias relative to distractor motion direction increases the longer participants remain in the same context, similarly for both clockwise and counterclockwise distractor contexts.

#### Relationship between search performance and probe deviation

Similarly, we found that the time since the switch block was also a significant predictor of search performance, with participants becoming better in the search task as time elapsed within a given distractor context (β = .178, *SE* = .014, *z* = 12.34, *p* < .001). Distractor condition was not a significant predictor of search performance (β = -.01, *SE* = .046, *z* = -.033, *p* = .743) and the two variables did not significantly interact (β = -.004, *SE* = .007, *z* = -.58, *p* = .559).

Finally, we found a significant relationship between probe deviation and search accuracy (Supplementary Figure 2). There was a positive correlation (r = .231, p < .001) between the two variables, indicating when the probe deviation was greater than search accuracy was also higher. Moreover, when the correlation coefficient is calculated per participant (across blocks) we find that the average correlation coefficient is significantly greater than zero (t_(53)_ = 7.42, p < .001, d = 1.01; M = .237, SD = .234)

## Discussion

The results of Experiment 2 replicate the observations in Experiment 1, extending them to a flexible setting in which the distractor context changes throughout the experiment. The findings reveal the flexible updating of functionally tuned dynamic attentional templates. Attentional templates were updated flexibly according to the current context. Interestingly, the bias grew over time within a context suggesting the contribution of a cumulative learning mechanism. The rate of this growth was not significantly affected by the context itself (clockwise vs. counterclockwise distractors). Interestingly, we find that performance on the search task was also affected by the time within a context. That is, as participants spend more time in each distractor context, their representation of the target becomes more biased. They also become more accurate in their search for the target. This mirrors the significant positive correlation that we found in both Experiments 1 & 2 and provides further evidence for a functional role of template biasing within dynamic templates.

This experiment pushes the notion of flexible dynamic attentional templates further, by demonstrating their flexibility. We found that participants biased their representations of motion direction away from a given distractor context in a volatile environment in which the distractor context regularly changed. In addition, the bias increased as the same context persisted. The spatial variability of the stimuli, the flexible reversal of the bias according to distractor motions, and the growing biases over consistent blocks argue against explanations based on low-level adaptation mechanisms, suggesting instead functional and adaptive tuning of templates guided by experience.

In both Experiments 1 and 2, motion direction was used as the only target-defining feature. Participants had to use motion direction to find the target, and a failure to do so would result in chance performance during the visual search. Outside of the laboratory, targets are often defined by multiple features. Although we may have explicit access to some of these features – I always know what my friend looks like – other features may have to be learned within the context of the task – I only know what she is wearing after an initial encounter. Past work has shown that we are quick to learn regularities within an environment and that these environmental regularities can guide our attention even when they are incidental to the task (Turk-Browne et al., 2005; Zhao et al., 2013). In Experiment 3, we asked whether participants learn and utilise a dynamic feature that is not explicitly instructed or required for identifying the target.

## Experiment 3: Biasing motion direction in multidimensional targets

### Methods

#### Transparency and Openness

We report details on the power analysis for which we based our sample size, data exclusion criteria, and all measures within the experiment. The data, analysis code, experimental code, and additional resources can be accessed https://osf.io/4mv9g/ (Boettcher & Nobre, 2023). The experiment was not preregistered.

#### Participants

A total of 175 participants were recruited via Prolific Academic (https://www.prolific.co/). After exclusion of six participants during pre-processing, 168 remained in the final analysis. Of those remaining, 84 participants were assigned to the clockwise distractors condition. They were between 19 and 40 years old (M = 27.17, SD = 5.63), 34 were female, 50 reported their gender as male, 71 were right-handed and 13 were left-handed. The other 84 participants were assigned to the counterclockwise distractors condition. They were between 19 and 40 years old (M = 27.95, SD = 5.81), 29 reported their gender as female, 55 reported their gender as male, 74 were right-handed, and 10 were left handed.

#### Task and Procedure

In Experiment 3 we tested whether the template biasing found in Experiments 1 and 2 relied on motion direction being the instructed defining target feature. To test this, the participants’ task was changed to finding the moving target dot defined by colour. The manipulations regarding the relative motion direction of distractors were preserved but discriminating motion was not required for performing the search task. At the start of the experiment, participants were shown an overview of the task, their target colour, and a video with examples of several trials, and then given an opportunity to practice.

**Figure 3:**
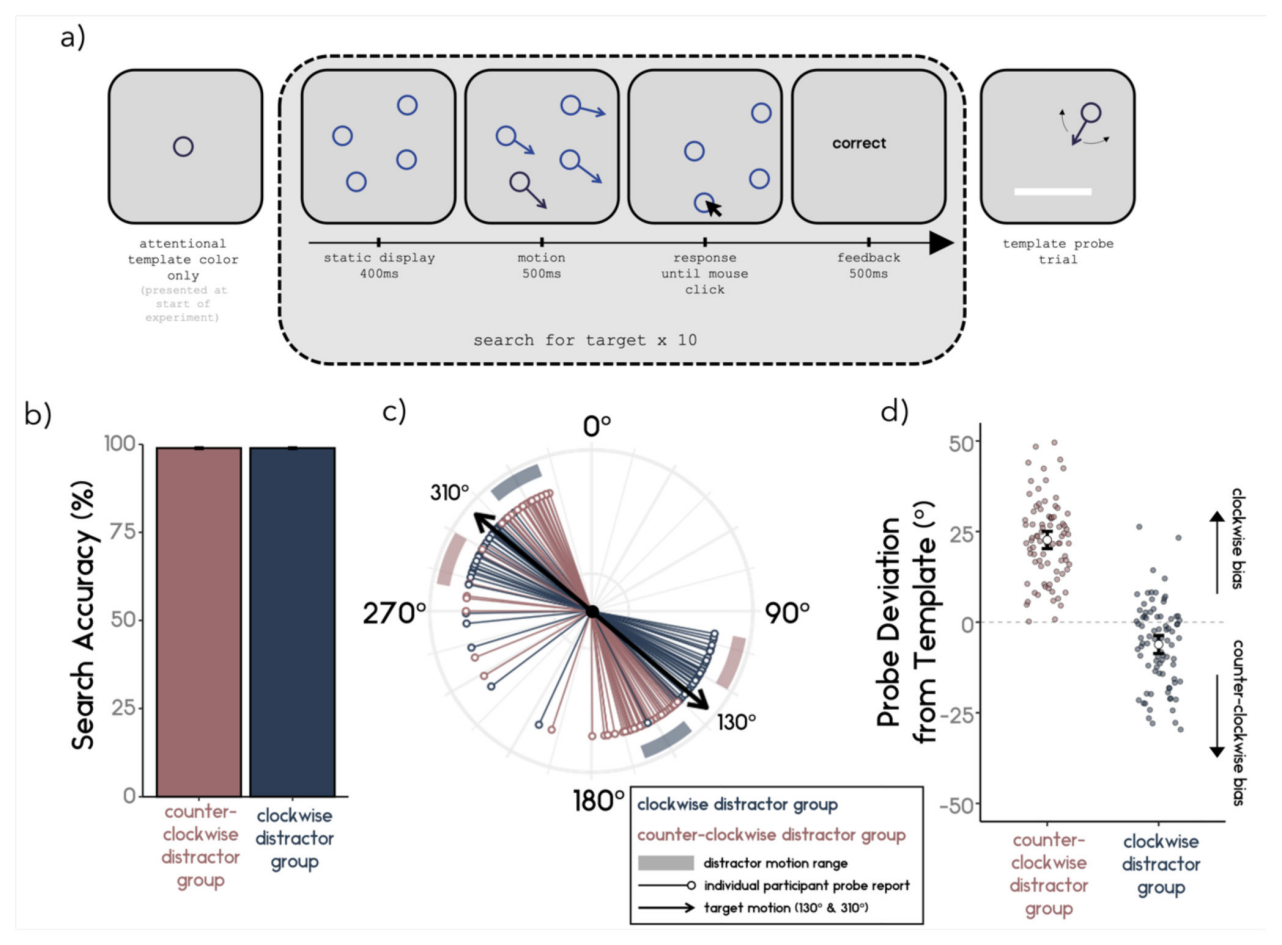
a) Schematic of Experiment 3. Participants were presented with the target colour at the start of the experiment. Participants searched for this colour among distractor dots. The target dot consistently moved in the same direction throughout the experiment, while the distractors moved either clockwise or counterclockwise relative to the target (balanced across participants). b) Participants’ search performance. Participants were near ceiling within this task, and accuracy across distractor conditions did not differ. c) Participant probe reports. Target motion was either 130° or 310° (black arrows) and the possible distractors encountered during the search were either clockwise (blueish shaded region) or counterclockwise (reddish shaded region) relative to the target motion. Participants’ average probe reports are plotted as thin coloured lines. Participants who encountered clockwise distractors on average reported their target as more counterclockwise, while those who encountered counterclockwise distractors reported the target as more clockwise. C) Average probe deviation from template. Individuals’ average deviation during probe trials from the true target motion direction for participants from the counterclockwise (red) and clockwise (blue) are plotted as small dots. Positive (negative) deviations indicate that the participant reported the probe as more clockwise (counterclockwise) than the target truly was. The average deviation for each group is represented as a white dot. Error bars represent the 95% confidence interval around the mean.

A summary of the experiment structure is shown in Figure 3a. Compared to Experiments 1 and 2, only the stimulus colour parameters changed. All stimulus motion and feedback parameters were the same. The stimulus colours started with a tealish blue outline colour (RGB: 47, 72, 155) and the same grey background as in previous experiments (RGB: 128, 128, 128). At the onset of motion, the outline of one stimulus changed to dark purply blue (RGB: 45, 42, 76) for the duration of motion and then returned to its original colour. The purplish colour defined the target item. The purplish item coincided with target motion in the 130° or 310° direction, but the direction of motion was a redundant feature and not required for target identification. After the stimuli stopped moving, participants clicked on the target and then received feedback onscreen.

The pattern of direction of motion was not made explicit to participants. Across conditions, the target stimuli moved in the 130° or 310° direction and distractors moved 10°, 20°, or 30° clockwise or counterclockwise to these. Like in Experiment 1, the distractor context was manipulated across participants. Half of the participants were exposed to clockwise distractors and the other half to counterclockwise distractors.

Participants completed ten experimental blocks. Each block consisted of ten search trials followed by a probe trial. In probe trials, participants were asked to report the motion direction of the target in the last individual search trial using a radial dial. The reporting dial and parameters were the same as in Experiments 1 and 2 and no feedback was provided.

#### Analysis

Data were pre-processed and cleaned using the same pipeline used in Experiments 1 and 2. Search trials with very slow or outlier RTs were removed. No participant was removed for loss of more than 10% of trials. No participant was removed for poor accuracy (< 50% correct). Probe trials were removed for very slow or outlier RTs. No participants were removed for discarding more than 20% of probe trials. Two participants were removed for probe deviations greater than 2.5 SD from the grand mean. During debrief, six participants indicated that they did not believe their data should be included. In total, six datasets were removed from the final sample resulting in a final sample size of 168 participants.

We calculated the average search performance across the two distractor conditions and checked whether they differed by conducting a Welch’s two-sample t-test. For the main analysis, the average deviation in the motion direction of the probe remained the dependent variable of interest. The main independent variable was distractor group (clockwise vs. counterclockwise), manipulated between participants. A Welch’s two sample *t-test* compared the average probe deviation between participants with clockwise vs. counterclockwise distractors. This was followed by two one-sample *t-tests* comparing the mean in probe deviation in each group against zero. All statistical tests were run using the *lsr package* in R (Navarro, 2015). *Cohen’s d* served as a measure of effect size.

Once again, to test the relationship between search accuracy and probe deviation we calculated each participant’s average search accuracy as well as their probe deviation in the corresponding block. Probe deviation was transformed such that positive values always indicate a high deviation no matter the distractor context. Like in the previous experiments, we tested for a significant correlation between these variables both across and within participants.

## Results

### Search Performance

Figure 3b *shows the average search accuracy for participants with counter- and clockwise distractors. The average search accuracy did not differ between the two groups (t*_(166.92)_ = 0.161, *p* = .872, *d* = .025; clockwise distractor group M = 98.91, SD = 1.08; counter-clockwise distractor group M = 98.94, SD = 1.02). Accuracy in this experiment was particularly high due to the nature of the search.

### Probe Deviation

Experiment 3 tested whether the distractor motion-direction context would still bias the representation of this non-compulsory, redundant target feature (Figures 3c & 3d). Distractor group had a significant effect on probe deviation (*t*_(165.67)_ = 16.64, *p* < .001, *d* = 2.56). The average probe deviation for the counterclockwise distractor group was biased in the clockwise direction (M = 22.64; SD = 10.97) (*t*_(83)_ = 18.93, *p* < .001, *d* = 2.06), while the average probe deviation for the clockwise distractor group was significantly biased in the counterclockwise direction (M = -6.16; SD = 11.47) (*t*_(83)_ = -4.92, *p* < .001, *d* = .54).

#### Relationship between search performance and probe deviation

In contrast to the previous experiments, we did not find a significant relationship between probe deviation and search accuracy (Supplementary Figure 2). This was not surprising given the minimal variability in the high levels of search performance. There was a non-significant relationship (r = -.0069, *p* = .78). Moreover, when the correlation coefficient is calculated per participant (across blocks) we find that the average correlation coefficient did not differ from zero (t_(103)_ = -.012, *p* =.991, *d* = .001; M = -0.0004, SD = .352).

## Discussion

Experiment 3 confirmed that the dynamic motion-direction attribute of the attentional template continued to be biased even when it did not exclusively define the target. The near-ceiling search performance in this experiment (compared to previous experiments) confirms that participants were likely using the colour information to perform the task. Although motion direction was not a necessary feature of the search, it remained useful for differentiating targets from distractors. Thus, learning the direction of motion of the target may provide a more complete attentional template, which, in turn, benefits behaviour (Schmidt & Zelinsky, 2017). A large and growing body of research suggests that participants automatically learn helpful regularities in their environment and use these to guide attention (Awh et al., 2012; Boettcher et al., 2021; Boettcher, Stokes, et al., 2020; Geng & Behrmann, 2002; Turk-Browne et al., 2005; Zhao et al., 2013). In this experiment, the relationship between the motion directions of the target and distractors was task-irrelevant. Nevertheless, we find a strong a consistent relationship between the distractor context and the report of the target motion direction. This suggests that participants are incidentally learning the useful regularities within the environment to guide behaviour.

In Experiment 3, we did not find a significant relationship between search performance and probe deviation. However, participants performed the search with near-perfect accuracy, with an average accuracy of 99%. For this reason, our metric of search performance – search accuracy – likely does not provide us with enough variability for detecting relationships between these variables. Future studies may wish to incorporate a design with which reaction time is a more meaningful variable and therefore could be used as an additional measure of search performance.

Motion direction is not the only dynamic feature available. Returning to our earlier example of searching for a friend in a pack of runners, we may have foreknowledge about our friend’s likely pace. Therefore, speed is another useful dynamic feature that can be incorporated into the attentional template. Testing whether motion speed can also be biased within the attentional template would speak to the generality of the ability to bias dynamic features in templates when functionally useful.

## Experiment 4: Biasing speed in dynamic attentional templates

## Methods

### Transparency and Openness

We report details on the power analysis for which we based our sample size, data exclusion criteria, and all measures within the experiment. The data, analysis code, experimental code, and additional resources can be accessed https://osf.io/4mv9g/ (Boettcher & Nobre, 2023). The experiment was not preregistered.

### Participants

A total of 223 participants were recruited for a final dataset of 168 in the full analysis (see *Data Preprocessing*). Participants were divided into two equal groups of 84 each. Participants in the slow-distractors condition (see below) were aged between 19 and 40 (M = 26.01, SD = 5.14). Thirty-four reported their gender as female, 50 reported their gender as male, 71 were right-handed, and 13 were left-handed. Participants in the fast-distractors condition were aged between 18 and 40 (M = 26.04, SD = 6.21). Twenty-eight reported their gender as female, 56 reported their gender as male, 79 were right-handed, and 5 were left-handed.

### Task and Procedure

**Figure 4:**
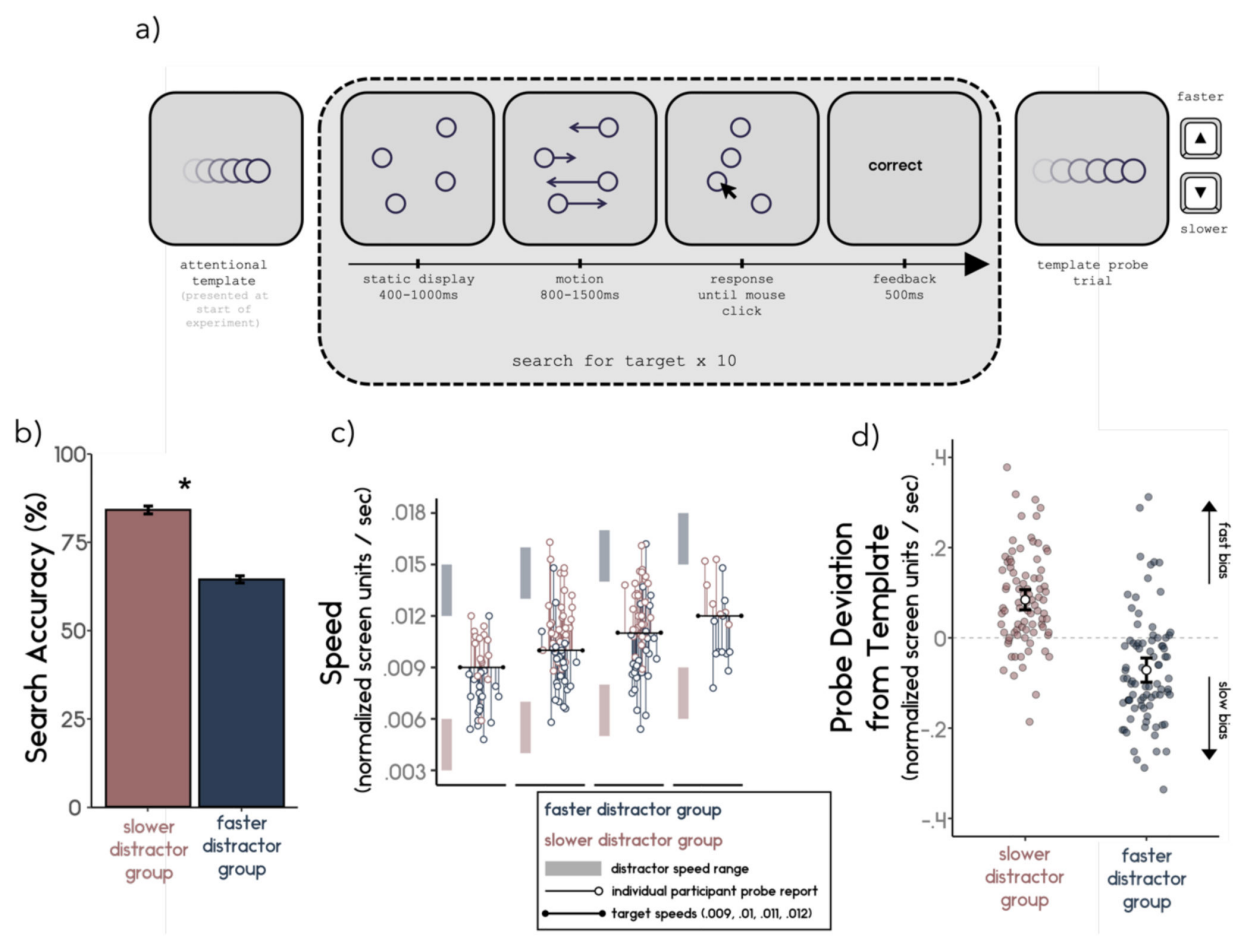
a) Schematic of Experiment 4. Participants were presented with the target speed at the start of the experiment. Participants searched for a dot moving at this speed among distractor dots. Distractors moved consistently faster or slower relative to the target (balanced across participants). b) Search performance. Participant performed significantly better in the search when searching for their target among slower distractors compared to faster distractors. c) Participant probe reports. Target speed was either .009, .01, .011, or .012 normalised screen units/sec (black lines) and the possible distractors encountered during the search were either faster (blueish shaded region) or slower (reddish shaded region) relative to the target speed. Participants’ average probe report are plotted as thin coloured lines. Participants who encountered faster distractors on average reported their target as slower, while those who encountered slower distractors reported the target as faster. d) Average probe deviation from template. Individuals’ average deviation during probe trials from the true target motion direction for participants from slower (reddish) and faster (blueish) distractor group are plotted as small dots. Positive (negative) deviations indicate that the participant reported the probe as faster (slower) than the target truly was. The average deviation for each group is represented as a white dot. Error bars represent the 95% confidence interval around the mean.

In Experiment 4, the participants’ task was to find a moving target dot defined by its speed of motion among distractor dots moving at different speeds. Figure 4a provides an overview of the task. At the start of the experiment, participants were shown an overview of the task and a video containing several example trials and provided with an opportunity to practice. Before the task began participants were shown their target speed (.009, .010, .011, or .012 normalised screen units/second). These were randomly assigned among participants. The target speed was presented to the participants as a target dot moving along the horizontal path in the middle of the screen (randomly presented either from left to right or right to left). The participant had to retain this speed in memory and use it for identifying targets throughout the experiment.

The stimulus parameters were identical to Experiments 1 and 2 with regards to colour and size but differed in the motion parameters. Motion duration as well as onset and offset times varied among stimuli to avoid speed discrimination being confounded with these. Each stimulus was randomly assigned a start time (between 400 and 1000 ms, in steps of 100 ms) as well as a movement duration (between 800 and 1500 ms, in steps of 100 ms). Stimuli moved along a horizontal path, and their motion direction was also randomly assigned (left to right or right to left). These random assignments were used to ensure that participants used *speed* of motion to find their targets rather than relying on onset or offset times, duration of motion, or spatial distance travelled for their responses.

Targets always moved at their predetermined speed (.009, .010, .011, or .012 normalised screen units/second). Distractors differed from the target speed by (.003, .0045, or .006 normalised screen units/second), consistently being either faster or slower. The exact combination of the three distractor speeds was chosen randomly with replacement on each trial. Half of the participants were assigned to the faster distractors group and were only exposed to distractors that were faster than the target (+.003, +.0045, +.006 normalised screen units/second). The other half of the participants were assigned to the slower distractors group and were only exposed to distractors that were slower than the target (-.003, -.0045, -.006 normalised screen units/second). After all the stimuli stopped moving, participants clicked on the target and then received feedback onscreen (either ‘correct’ or ‘incorrect’) for a 500-ms period. After a random delay between 300 and 800 ms, the next trial began.

Participants completed ten experimental blocks. Each block consisted of ten search trials followed by a probe trial. In probe trials, participants reported the speed of their target. Participants were presented with a stimulus moving either to the left or the right along the horizontal plane. The probe stimulus was randomly assigned a start speed ±.009 (in steps of .001) normalised screen units/second from the target speed. For example, if a participant’s target speed was .011 normalised screen units/second, the probe could start at any speed (in steps of .001) between .002 and .02 normalised screen units/second. Participants then adjusted the probe by using the up and down arrow keys to make the dot move faster and slower, respectively. Once participants were satisfied that the probe was moving at the same speed as their target, they pressed the space bar to confirm their response. There was no time limit for reporting the speed and there was no feedback on probe trials.

### Analysis

Data analyses was carried out in R Studio (RStudio Team, 2019). Data were first pre-processed and cleaned using a similar pipeline to Experiments 1-3. Specifically, slow search trials were initially removed from the data set. That is, trials in which the RT exceeded 5000 ms or was 2.5 SD above the individual mean. Participants with more than 10% slow searches were removed from the analysis, resulting in the loss of three datasets. Next, participants who performed poorly on the search (< 50% correct) were also removed. This resulted in the loss of 47 datasets. Probe trials in which the probe deviation was more than 2.5 SD from the participant’s mean were removed. A cut-off for discarding participants with more than 20% of probe trials discarded did not result in the loss any additional datasets. Four participants were discarded for having an overall probe deviation greater than 2.5 SD from the grand mean. Debriefing resulted in four participants indicating that they did not believe their data should be included. In total 55 datasets were removed resulting in a final sample size of 168 participants.

We first checked whether search performance differed between the two distractor conditions by conducting a repeated measures *t-test* comparing the average search performance for slower and faster distractor groups.

Like the previous experiments, our main analysis of interest concerned the probe trials. The dependent variable of interest, average probe speed deviation, was calculated as the difference between the reported speed during probe trials and the true target speed. Average probe deviation was calculated per participant and collapsed across the four possible target speeds. An average deviation of zero would indicate no bias. An average positive deviation would indicate a bias for representing the target as faster than the actual target speed, whereas an average negative deviation would indicate a bias slower than the actual target speed. Again, the main independent variable was distractor group (faster vs. slower distractors), manipulated between participants. Our main analysis consisted of a Welch’s two sample *t-test* comparing the average probe deviation for participants with faster vs. slower distractors. This was followed by two one-sample *t-tests* comparing the mean in probe deviation in each group against zero. All statistical tests were run using the *lsr package* in R (Navarro, 2015). *Cohen’s d* provides a measure of effect size.

Finally, to test the relationship between search accuracy and probe deviation we again calculated each participant’s average search accuracy as well as their probe deviation in the corresponding block. Probe deviation was transformed such that positive values always indicate a high deviation no matter the distractor context. Like in the previous experiments, we tested for a significant correlation between these variables both across and within participants.

## Results

### Search Performance

Figure 4b *shows the average search accuracy for participants in the faster and slower distractor groups. Participants in the slower distractor group (M=83.99, SD = 10.58) were significant better at finding their target compared to participants in the faster distractor group (M = 64.32, SD = 9.11) (t*_(167.38)_ = -13.07, *p* < .001, *d* = 1.99.

### Probe Deviation

Distractor group had a significant effect on probe deviation (Figures 4b and 4c; *t*_(160.94)_ = 8.86, *p* < .001, *d* = 1.27). The average probe speed reported was significantly biased in both slower and faster distractor groups. For the slower distractor group, the probe speed was reported as significantly faster than the true target speed (M = .084; SD = .103) (*t*_(83)_ = 7.49, *p* < .001, *d* = .82), whereas the probe speed was reported to be slower than the true target speed for the faster distractor group (M = -.071; SD = .124) (*t*_(83)_ = -5.29, *p* < .001, *d* = .58).

### Relationship between search performance and probe deviation

Again, we found a significant positive correlation between probe deviation and search accuracy (r = .064, p = .0086) (Supplementary Figure 2). Search performance improved as the probe deviation was greater. Moreover, when the correlation coefficient is calculated per participant (across blocks) we find that the average correlation coefficient is significantly greater than zero (t_(167)_ = 2.42, p =.016, d = .187; M = .06, SD = .323).

## Discussion

The results of Experiment 4 complement the results of Experiments 1-3 by demonstrating the ability to adapt dynamic target representations based on motion speed. Participants consistently reported target speeds that were biased away from the distractor context. Participants exposed to distractors faster than the target reported the target as being slower than it truly was. Participants exposed to distractors slower than the target reported the target as being faster than it was. Our correlation analysis showed that this biasing was again related to search performance.

Interestingly, we observe a difference in search performance depending on whether participants search for targets among relatively slower of faster distractors. Performance was reliably better for searching for a faster target among slower distractors. This asymmetry has been found previously and may reflect an evolutionary bias towards fast moving stimuli (Ivry & Cohen, 1992).

Extending the observation of functional biasing of dynamic attributes from motion direction to motion speed suggests a general biasing mechanism that can integrate sensory signals over time even when there is no clear spatial anchor to guide effective visual search in dynamic contexts.

## General Discussion

Across four experiments we tested whether representations of dynamic stimulus attributes became functionally biased by their context. By directly probing the memory for motion features of targets following trials of a dynamic visual search task, we found converging evidence that memory representations of motion direction and speed systematically diverged from the veridical to guide behaviour. Moreover, we found that this biasing was consistently related to improvements in search performance within and across participants.

In Experiments 1 and 2, participants searched for a target defined by direction of motion. In both experiments, participants then reported the direction of motion of their target as being biased away from the motion directions of competing distractors. That is, when searching among distractors that moved clockwise relative to the target, the target was reported as being more counterclockwise. The complementary bias occurred when searching among distractors that moved counterclockwise. Experiment 2 revealed flexibility in this effect, as the relative motion direction of competing distractors repeatedly switched over the course of the task. The biasing of the target motion direction grew as participants spent more time in each distractor context. Interestingly, Experiment 3 showed that motion-direction biases formed even when this attribute was redundant and not required to identify the target, which was specified according to its colour, a non-dynamic feature. Although the relationship between the motion direction of the target and distractors was task-irrelevant during search, participants nevertheless reported the target motion direction as biased away from the distractor directions in probe trials, indicating they incidentally learned the relation between target and distractor motion directions during search. Finally, Experiment 4 extended the finding of biases in dynamic stimulus features to motion speed, when the stimuli had no clear spatial anchor and began moving with variable onsets and durations.

In Experiments 1, 2, and 4 we found evidence for a positive relationship between search performance and template bias. That is, when participants reported the target as more biased (i.e., further away from the distractor values) they also performed better in the search task. This relationship was not present in Experiment 3, in which search performance reached ceiling and may thus not provide a sensitive measure. We found further evidence for a connection between search performance and template biasing in Experiment 2. Here, we saw that both template biasing and search performance increase as a function of elapsed time in a consistent distractor context. These results show a strong a relationship between behavior during search and on memory probe trials. This suggests a functional benefit to biases within the attentional template.

Beyond templates for visual search, biases have also been found in working-memory tasks. The serial dependence literature has provided compelling evidence that the reported working-memory representation in the current trial is systematically influenced by information from the previous trial, even though this is no longer relevant (C. Fischer et al., 2020; J. Fischer & Whitney, 2014; Hajonides et al., 2023). Researchers have suggested that both repulsive and attractive biases within working memory can be adaptive (Brady & Alvarez, 2011; Chunharas et al., 2022; Hajonides et al., 2023; Scotti, Hong, Leber, et al., 2021). Attractive biases towards a particular value may reflect an integration of group-level statistics, while repulsive biases may reflect a means for distinguishing items in memory. Consistent with this view, we observed participants’ memory representations to be biased away from the distractors, which could help distinguish relevant from competing the stimuli.

In Experiments 1-3, we consistently find a stronger bias in the clockwise direction. It is important to note that within our design these asymmetries cannot be attributed to attentional difference in the vertical or horizontal direction alone. This is because we utilised two target directions, 180° apart. For half our participants, clockwise relative to the target is ‘down and to the left’, while for the other half of participants clockwise is ‘up and to the right’. We speculate that this asymmetry may reflect an inherent bias towards the clockwise direction (and therefore away from counterclockwise distractors). This clockwise (relative to counterclockwise) asymmetry is not unique to our task and stimulus parameters. It has been noted in other areas of psychology and neuroscience (for a review see: Karim et al., 2016), such as in perception (Lucafò et al., 2016; Troje & McAdam, 2010) and action in both human (Stochl & Croudace, 2013) and non-human animals (Lucky et al., 2012; MacNeilage, 2014; Schwarting & Borta, 2005). Our data fit with these past findings and show a stronger bias in the clockwise direction. However, importantly, we also find a significant bias in the counterclockwise direction showing that template biasing occurs above and beyond these natural baseline asymmetries.

The exact mechanisms through which attentional templates become biased are still debated, with two alternative explanations prevailing (Yu et al., 2023). Some researchers suggest that items in memory are biased because of low-level perceptual contrasting effects between targets and distractors (Hamblin-Frohman & Becker, 2021). In this case, the target-relevant feature is perceived and subsequently encoded in a skewed manner within the context of the distractors. Past work has shown biases of static features in perceptual tasks(Chapman et al., 2023; Chen et al., 2019; MacLeod, 2003; Störmer & Alvarez, 2014). These effects extend to dynamic stimuli. For example, perception of motion direction has been reported to be biased away from reference motion (or line) – an effect known as *reference repulsion* (Kim & Wilson, 1997; Marshak & Sekuler, 1979; Zamboni et al., 2016). In the current experiments, although no explicit reference line was provided, a distortion of participants’ *perception* of the target motion could have contributed to the reported biases in the attentional templates.

As an alternative, researchers have proposed that memory representations become biased to maximise their functional utility for distinguishing between the target and expected distractors (Geng & Witkowski, 2019). Evidence for this account is growing. Functional biases have been observed for targets retrieved from associative long-term memory even without any distractors present (Boettcher, van Ede, et al., 2020). In this previous report (Boettcher, van Ede, et al., 2020), participants learned to associate shapes with coloured gratings. In a subsequent task they viewed a shape followed by a grating and indicated if the stimuli matched the learned association. Participants were found to prioritise either the colour or orientation of the grating depending on whether non-matching gratings in lure pairs were more likely to share colour or orientation. They were better and faster to exclude non-match gratings with diagnostic features. Differential feature weighting within the associated attentional templates showed flexible biasing in a design that precluded low-level sensory-biasing effects, with individual distractor features fully matched within conditions. Moreover, recent studies have reported that template biases are modulated by task demands, confirming functional adaptations that cannot be explained entirely by sensory parameters (Yu et al., 2022). In the current experiments, the bias effect grew with continued use of the template, suggesting a functional contribution of learning that beneficially helped distinguish targets from distractors.

In principle, the two accounts are not mutually exclusive and could even work together in a mutually supportive way. The contrastive properties of sensory processing during visual perception themselves likely evolved for their utility for identifying and discriminating items in the environment. Thus, intrinsic sensory distortions may prove beneficial for visual search performance when contrastive properties help distinguish targets from distractors with systematically competing sensory attributes. Encoding into memory necessarily builds on products of perception, so representations will carry the resulting intrinsic distortions. However, additionally, memory representations may undergo further modulation to maximise their value in serving task goals. The growing demonstrations of the flexibility and pliability of memory content according to task goals and expectations support the existence of post-perceptual mechanisms that can adapt and bias memory states to guide behaviour (Nobre & Stokes, 2019; Van Ede & Nobre, 2023; Yu et al., 2023).

Our study was not aimed at arbitrating between the origin(s) of template biases. Instead, the focus was on demonstrating biasing of *dynamic* attributes within attentional templates. The confirmation of biasing of dynamic templates implies mechanisms that can integrate signals over time to learn and utilise effective templates. Across four experiments, memory representations of motion direction and speed were biased from their true form. Having established the possibility of biasing dynamic attributes, future studies can investigate the mechanisms. We speculate that both perceptual and memory-specific mechanisms work in tandem to guide efficient behaviour. In the current set of experiments, perceptual distortions from sensory processing during the visual search may be encoded and start to overwrite the memory representation of the explicitly trained target. The resulting biased representation can, in turn, guide attention and decision-making during subsequent search. In the context of distractors with systematic variation in competing features, the biased template is indeed functional and better for identifying targets compared to the veridical target representation. As such, participants do not need to correct the perceptual biases towards veridical but rather may continue push their target representation away from the distractors (as suggested by the increasing bias in Experiment 2). Future work may wish to interrogate the utility of these biases during visual search by introducing ‘catch’ trials in which distractors are unexpectedly symmetrical. If the biasing is truly functional, participants should be worse at finding the target in these trials.

Beyond the low-level vs. functional origin of template biases, the literature on static attentional templates has also considered the shape of the template modulation. The ‘optimal tuning’ account proposes that the target-defining feature value is shifted in the template, with the tuning around this new feature remaining normal (Navalpakkam & Itti, 2007; Yu & Geng, 2019). For example, when searching for targets moving along 130° among clockwise distractors, the template may shift to represent a counterclockwise direction (e.g., 120°). An alternative ‘relational’ account proposes that attention is directed to the relationship between a target and its distractors, without the need for a shift within the template representation (Becker et al., 2010, 2013; Hamblin-Frohman & Becker, 2021). When searching for targets moving along 130° among clockwise distractors, participants look for the most counterclockwise item. The current findings cannot adjudicate between these accounts as our stimulus parameters supported the use of either. Future studies may wish to prevent this by including distractors from both sides of the distribution with a consistent skew (e.g., clockwise and counterclockwise distractors which are on average mostly clockwise). Nevertheless, both accounts have been theorised to contribute to attentional allocation at various stages within the search process (Yu et al., 2023).

The current work provides another interesting distinction from past work with static templates. The transient nature of the target-defining feature implies that the biased template must either be available at early stages of search or that the search display itself must be encoded into memory for further interrogation. The latter would necessitate participants holding the four search stimuli in working memory alongside their target template. This pushes the known limits of human memory. Therefore, it is much more likely that the biased template is implemented from the earliest stages of search (Yu et al., 2023).

This insight into the temporal dynamics of the utility of a biased template is only possible when a target feature is transient and dynamic, as is the case with moving stimuli.

Neural measures would help reveal when and how representations become biased. Representations held in memory proactively influence sensory neurons to modulate ongoing behaviour (Desimone & Duncan, 1995).However, it remains unclear whether the contents in memory representations need to overlap fully with the information that ultimately guides attention. Biased attentional templates may derive from similarly biased memory representations or from the flexible modulation of unbiased memory representations during their retrieval or output gating. Past work has shown that veridical information held in long-term associative memory may be selectively biased when it is used to guide behaviour (Boettcher, van Ede, et al., 2020). This indicates that veridical memory stores may be selectively biased upon retrieval when it is functional to do so. Future work aimed at disentangling what is *stored* vs. what is *used* is important for furthering our understanding of the interactions between memory and attention.

Taken together the current work provides an important step forward in our understanding of the interactions between memory and attention. Specifically, we provided convincing evidence that memory-derived dynamic attentional templates need not be veridical. Motion attributes become systematically and significantly biased when functionally adaptive for guide visual search in extended contexts. The findings imply the existence of biasing mechanisms that integrate over time and space to tune the formation or utilisation of memory-based templates to drive effective and flexible performance. This flexible biasing system holds important implications for how attention unfolds dynamically in time – introducing an important shift away from static models of attention.

### Constraints on Generality

The present study examined biases within memory representations which are used to guide visual search. The demographics of our cohort constrain interpretations regarding the generality of our findings. We tested healthy young, English-speaking adults under the age of forty. It is well established that both memory (Alloway & Alloway, 2013) and attention (Hommel et al., 2004) vary across the lifespan. This is also true for dynamic tasks (Shalev et al., 2022). As such, it is possible that the biases found within our sample of predominantly young adults may not generalise to all age groups. Moreover, although our sample of online participants allows a more global reach compared to in-person testing, the participant pool is still predominantly Western. This is particularly true because of our requirement for fluent English to ensure comprehension of task instructions.

## Supplementary Material

SuppFiles

## Impact Statement

This study shows that attentional templates for dynamic stimulus contents need not be veridical. Instead, across four experiments, we find that dynamic templates guiding visual search are consistently biased away from the distractors within the environment. The biasing of dynamic features is non-trivial, requiring learning mechanisms that can abstract over space and time and guidance mechanisms that can operate flexibly within unfolding contexts.

## Public Significance Statement

Our memories play a vital role in anticipating events in the world. Contrary to our intuitions, the memories that guide our anticipatory attention are not always truthful representations of the past. Instead, memory representations that guide attention as we search through the visual world can be distorted to emphasize the differences between the items that are important to us and other distracting items that may co-occur. To date, the biasing of these ‘attentional templates’ has only been demonstrated for static stimulus features. Here, we demonstrate that biasing also impacts dynamic stimulus features. This is important since we behave within dynamic contexts. Upgrading the biasing of attentional templates from static to dynamic features is non-trivial, requiring the brain to abstract over space and time to learn about and use the most useful information to guide adaptive behaviour.

## References

[R1] Alloway TP, Alloway RG (2013). Working memory across the lifespan: A cross-sectional approach. Journal of Cognitive Psychology.

[R2] Awh E, Belopolsky AV, Theeuwes J (2012). Top-down versus bottom-up attentional control: a failed theoretical dichotomy. Trends in Cognitive Sciences.

[R3] Bae GY, Luck SJ (2017). Interactions between visual working memory representations. Attention, Perception, and Psychophysics.

[R4] Barr DJ, Levy R, Scheepers C, Tily HJ (2013). Random effects structure for confirmatory hypothesis testing: Keep it maximal. Journal of Memory and Language.

[R5] Bates D, Mächler M, Bolker BM, Walker SC (2015). Fitting Linear Mixed-Effects Models Using lme4. Journal of Statistical Software.

[R6] Becker SI, Folk CL, Remington RW (2010). The Role of Relational Information in Contingent Capture. Journal of Experimental Psychology: Human Perception and Performance.

[R7] Becker SI, Folk CL, Remington RW (2013). Attentional Capture Does Not Depend on Feature Similarity, but on Target-Nontarget Relations. Psychological Science.

[R8] Boettcher SEP, Nobre AC (2023). Dynamic Template Biasing.

[R9] Boettcher SEP, Shalev N, Wolfe JM, Nobre AC (2021). Right place, right time: Spatiotemporal predictions guide attention in dynamic visual search. Journal of Experimental Psychology: General.

[R10] Boettcher SEP, Stokes MG, Nobre AC, van Ede F (2020). One thing leads to another: anticipating visual object identity based on associative-memory templates. The Journal of Neuroscience.

[R11] Boettcher SEP, van Ede F, Nobre AC (2020). Functional biases in attentional templates from associative memory. Journal of Vision.

[R12] Brady TF, Alvarez GA (2011). Hierarchical Encoding in Visual Working Memory.

[R13] Carlisle NB, Arita JT, Pardo D, Woodman GF (2011). Attentional templates in visual working memory. Journal of Neuroscience.

[R14] Chapman AF, Chunharas C, Störmer VS (2023). Feature-based attention warps the perception of visual features. Scientific Reports.

[R15] Chen J, Leber AB, Golomb JD (2019). Attentional Capture Alters Feature Perception. Journal of Experimental Psychology: Human Perception and Performance.

[R16] Chunharas C, Rademaker RL, Brady TF, Serences JT (2022). An Adaptive Perspective on Visual Working Memory Distortions. Journal of Experimental Psychology: General.

[R17] Desimone R, Duncan J (1995). Neural mechanisms of selective visual attention. Annual Review of Neuroscience.

[R18] Driver J, McLeod P, Dienes Z (1992). Are direction and speed coded independently by the visual system? Evidence from visual search. Spatial Vision.

[R19] Duncan J, Humphreys GW (1989). Visual search and stimulus similarity. Psychological Review.

[R20] Faul F, Erdfelder E, Lang AG, Buchner A (2007). G*Power 3: A flexible statistical power analysis program for the social, behavioral, and biomedical sciences. Behavior Research Methods.

[R21] Fischer C, Czoschke S, Peters B, Rahm B, Kaiser J, Bledowski C (2020). Context information supports serial dependence of multiple visual objects across memory episodes. Nature Communications.

[R22] Fischer J, Whitney D (2014). Serial dependence in visual perception. Nature Neuroscience.

[R23] Geng JJ, Behrmann M (2002). Probability cuing of target location facilitates visual search implicitly in normal participants and patients with hemispatial neglect. Psychological Science.

[R24] Geng JJ, Witkowski P (2019). Current Opinion in Psychology.

[R25] Hajonides JE, van Ede F, Stokes MG, Nobre AC, Myers NE (2023). Multiple and Dissociable Effects of Sensory History on Working-Memory Performance. Journal of Neuroscience.

[R26] Hamblin-Frohman Z, Becker SI (2021). The attentional template in high and low similarity search: Optimal tuning or tuning to relations?. Cognition.

[R27] Hommel B, Li KZH, Li SC (2004). Visual search across the life span. Developmental Psychology.

[R28] Horowitz TS, Wolfe JM, DiMase JS, Klieger SB (2007). Visual search for type of motion is based on simple motion primitives. Perception.

[R29] Ivry RB, Cohen A (1992). Asymmetry in Visual Search for Targets Defined by Differences in Movement Speed. Journal of Experimental Psychology: Human Perception and Performance.

[R30] Karim AKMR, Proulx MJ, Likova LT (2016). Anticlockwise or clockwise? A dynamic Perception-Action-Laterality model for directionality bias in visuospatial functioning. Neuroscience and Biobehavioral Reviews.

[R31] Kerzel D (2020). Direct Evidence for the Optimal Tuning of Attention. Journal of Experimental Psychology: Human Perception and Performance.

[R32] Kim J, Wilson HR (1997). Direction repulsion between components in motion transparency. Ophthalmic Literature.

[R33] Kuznetsova A, Brockhoff P, Christensen R (2017). lmerTest Package: Tests in Linear Mixed Effects Models. Journal of Statistical Software.

[R34] Lucafò C, Marzoli D, Prete G, Tommasi L (2016). Laterality effects in the spinning dancer illusion: The viewing-from-above bias is only part of the story. British Journal of Psychology (London, England : 1953).

[R35] Lucky NS, Ihara R, Yamaoka K, Hori M (2012). Behavioral Laterality and Morphological Asymmetry in the Cuttlefish, Sepia lycidas.

[R36] MacLeod DIA (2003). New dimensions in color perception. Trends in Cognitive Sciences.

[R37] MacNeilage PF (2014). Evolution of the strongest vertebrate rightward action asymmetries: Marine mammal sidedness and human handedness. Psychological Bulletin.

[R38] Marshak W, Sekuler R (1979). Mutual Repulsion Between Moving Visual Targets. Science.

[R39] Navalpakkam V, Itti L (2007). Search Goal Tunes Visual Features Optimally. Neuron.

[R40] Navarro DJ (2015). Learning statistics with R : a tutorial for psychology students and other beginners.

[R41] Nobre AC, Stokes MG (2019). Premembering Experience: A Hierarchy of Time-Scales for Proactive Attention. Neuron.

[R42] Nobre AC, van Ede F (2017). Anticipated moments: temporal structure in attention. Nature Reviews Neuroscience.

[R43] Nobre AC, van Ede F (2023). Attention in flux. Neuron.

[R44] Palan S, Schitter C (2018). Prolific.ac—A subject pool for online experiments. Journal of Behavioral and Experimental Finance.

[R45] Peirce J, Gray JR, Simpson S, MacAskill M, Höchenberger R, Sogo H, Kastman E, Lindeløv JK (2019). PsychoPy2: Experiments in behavior made easy. Behavior Research Methods.

[R46] Sauter M, Draschkow D, Mack W (2020). Building, Hosting and Recruiting: A Brief Introduction to Running Behavioral Experiments Online. Brain Sciences.

[R47] Schmidt J, Zelinsky GJ (2017). Adding details to the attentional template offsets search difficulty: Evidence from contralateral delay activity. Journal of Experimental Psychology: Human Perception and Performance.

[R48] Schwarting RKW, Borta A (2005). Analysis of behavioral asymmetries in the elevated plus-maze and in the T-maze. Journal of Neuroscience Methods.

[R49] Scolari M, Serences JT (2009). Adaptive allocation of attentional gain. Journal of Neuroscience.

[R50] Scotti PS, Hong Y, Golomb JD, Leber AB (2021). Statistical learning as a reference point for memory distortions: Swap and shift errors. Attention, Perception, and Psychophysics.

[R51] Scotti PS, Hong Y, Leber AB, Golomb JD (2021). Visual Working Memory Items Drift Apart Due to Active, Not Passive, Maintenance. Journal of Experimental Psychology: General.

[R52] Shalev N, Boettcher S, Wilkinson H, Scerif G, Nobre AC (2022). Be there on time: Spatial-temporal regularities guide young children’s attention in dynamic environments. Child Development.

[R53] Stochl J, Croudace T (2013). Predictors of human rotation. Laterality: Asymmetries of Body, Brain and Cognition.

[R54] Störmer VS, Alvarez GA (2014). Feature-Based Attention Elicits Surround Suppression in Feature Space. Current Biology.

[R55] Troje NF, McAdam M (2010). The viewing-from-above bias and the silhouette illusion. I-Perception.

[R56] Turk-Browne NB, Jungé JA, Scholl BJ (2005). The Automaticity of Visual Statistical Learning. Journal of Experimental Psychology: General.

[R57] Van Ede F, Nobre AC (2023). Turning Attention Inside Out: How Working Memory Serves Behavior. Annual Review of Psychology.

[R58] Wolfe JM (2021). Guided Search 6.0: An updated model of visual search. Psychonomic Bulletin and Review.

[R59] Yu X, Geng JJ (2019). The attentional template is shifted and asymmetrically sharpened by distractor context. Journal of Experimental Psychology: Human Perception and Performance.

[R60] Yu X, Rahim RA, Geng JJ (2022). Task-adaptive changes to the target template in response to distractor context: separability versus similarity. PsyArXiv.

[R61] Yu X, Zhou Z, Becker SI, Boettcher SEP, Geng JJ (2023). Good-enough attentional guidance. Trends in Cognitive Sciences.

[R62] Zamboni E, Ledgeway T, McGraw PV, Schluppeck D (2016). Do perceptual biases emerge early or late in visual processing? Decision-biases in motion perception. Proceedings of the Royal Society B: Biological Sciences.

[R63] Zhao J, Al-Aidroos N, Turk-Browne NB (2013). Attention Is Spontaneously Biased Toward Regularities. Psychological Science.

