# Supplementary material for: Going through the motions: biasing of dynamic attentional templates
Source: J Exp Psychol Gen. Author manuscript; Available in PMC 2025 Jul 7. (PMC7617878; doi:10.1037/xge0001665)
Supplement: SuppFiles [file EMS206499-supplement-SuppFiles.docx]

**Supplementary Figures**


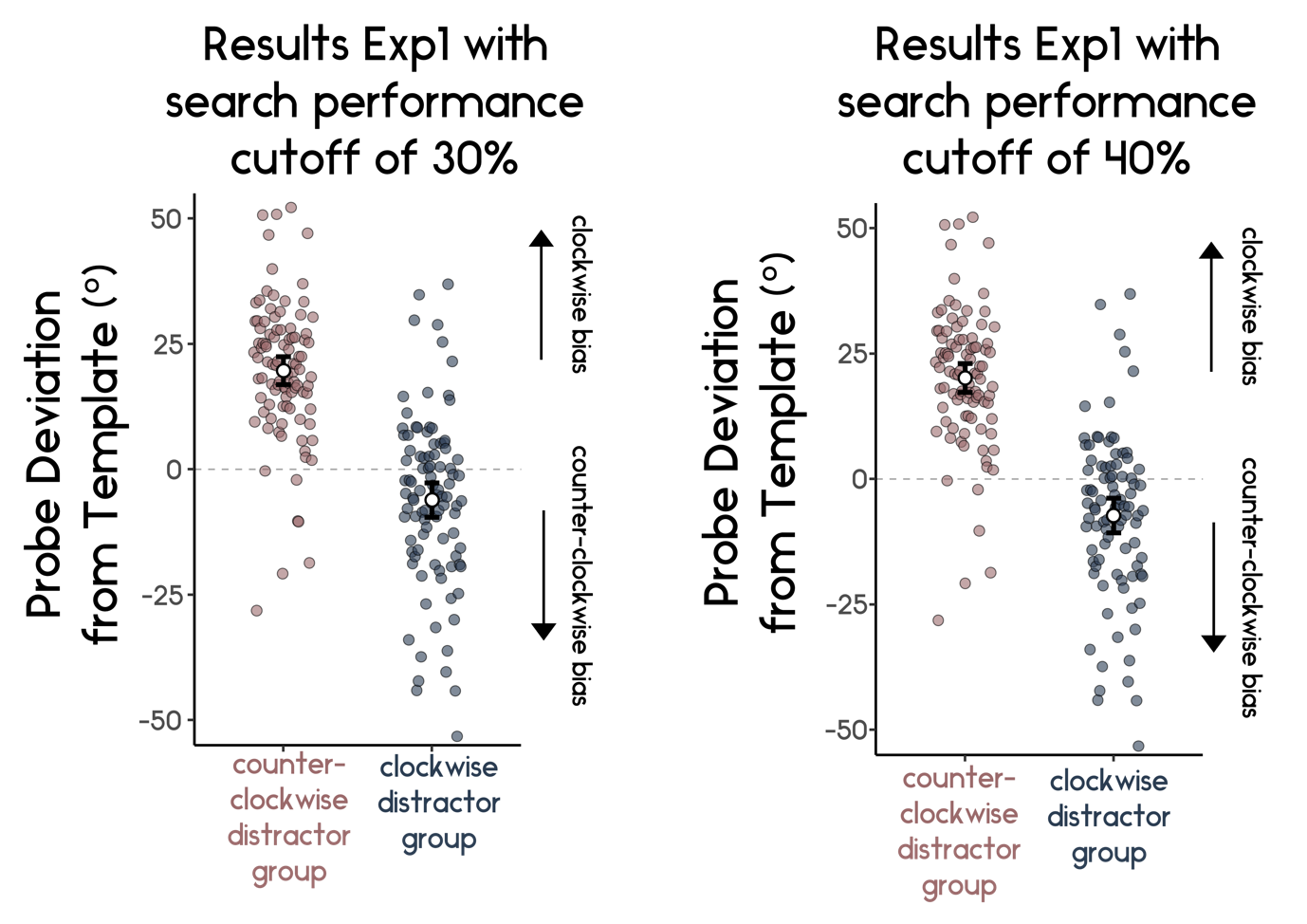


Supplementary Figure 1: Our results hold with more liberal data cleaning procedures, within the main analysis we used a predefined cut-off of 50% in the search task. Here the results stay largely the same with a cut-offs of 30% or 40%.

*
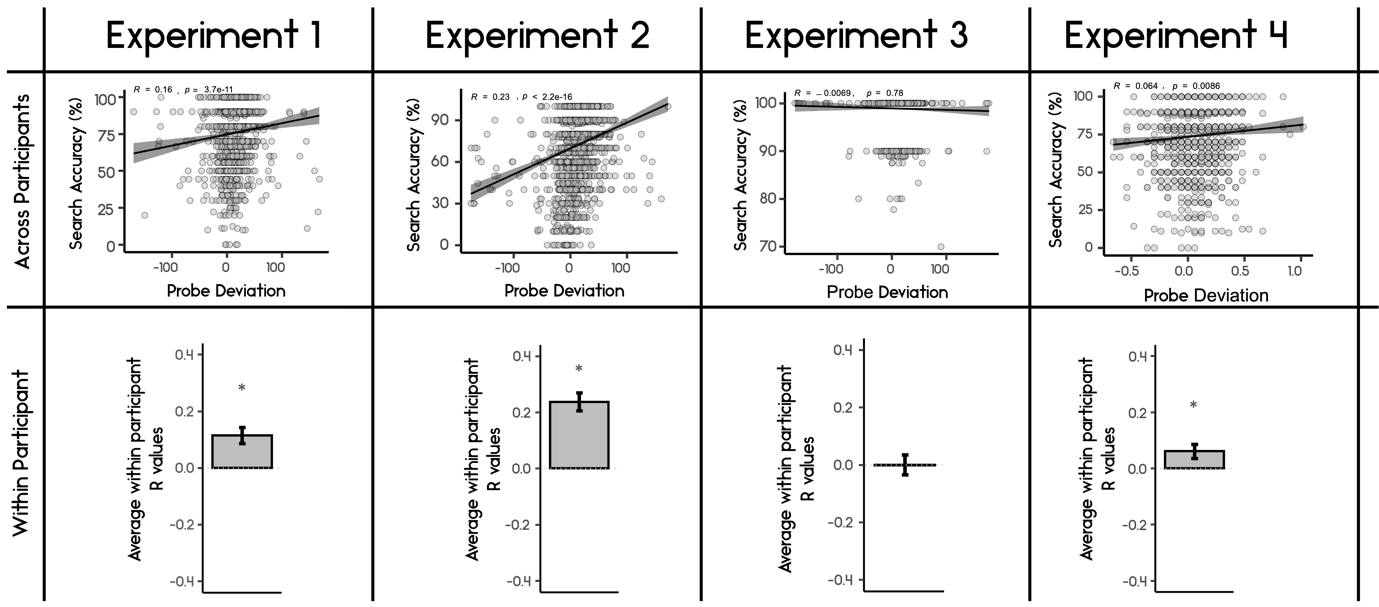
*

***Supplementary Figure 2****: Relationship between Search accuracy and Probe Deviation across the four experiments. Search Accuracy and Probe Deviation showed a significant positive correlation in all experiments except for Experiment 3 where Search Accuracy was at ceiling.*
